# Exploring fungal RiPPs from the perspective of chemical ecology

**DOI:** 10.1186/s40694-022-00144-9

**Published:** 2022-06-25

**Authors:** R. E. Ford, G. D. Foster, A. M. Bailey

**Affiliations:** School of Biological Sciences, University of Bristol, Life Sciences Building, 28 Tyndall Ave, Bristol, BS8 1TQ UK

**Keywords:** RiPP, Chemical ecology, Secondary metabolites, Mycotoxin

## Abstract

Since the initial detection, in 2007, of fungal ribosomally synthesised and post-translationally modified peptides (RiPPs), this group of natural products has undergone rapid expansion, with four separate classes now recognised: amatoxins/phallotoxins, borosins, dikaritins, and epichloëcyclins. Largely due to their historically anthropocentric employment in medicine and agriculture, novel fungal proteins and peptides are seldom investigated in relation to the fungus itself. Therefore, although the benefits these compounds confer to humans are often realised, their evolutionary advantage to the fungus, the reason for their continued production, is often obscure or ignored. This review sets out to summarise current knowledge on how these small peptide-derived products influence their producing species and surrounding biotic environment.

## Ribosomally synthesised and post-translationally modified peptides (RiPPs)

**Fig. 1:**
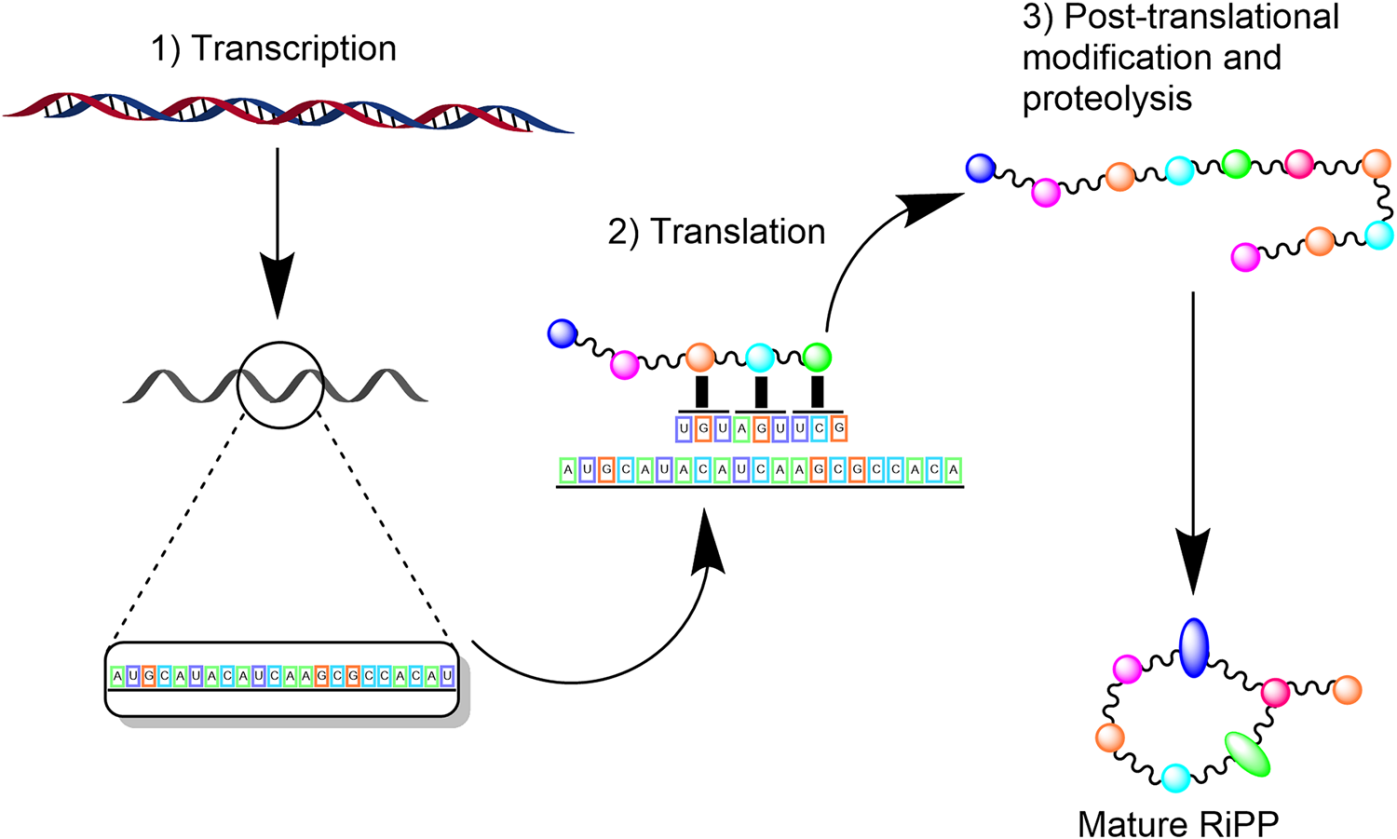
Illustration of post-ribosomal peptide synthesis. The fungal biosynthetic gene cluster is transcribed and translated to give a precursor protein which then undergoes post-translational modification and proteolysis to give the final mature RiPP

Fungi, or more specifically small molecules from fungi, have become indispensable in human lives through their employment in medicine, treating infection (penicillin: [1, 2]) and reducing the risk of disease (lovastatin: [3]). Absence or loss of these compounds would challenge current medical procedures and may result in reduced human lifespans. Fortunately, fungi are famous producers of a significant array of secondary metabolites, with continual exploration of these compounds permitting novel drug discovery and alleviating these concerns. Following the realisation that penicillin, cephalosporin and cyclosporines were produced by non-ribosomal peptide synthetases (NRPS), small fungal peptides have usually been assumed to be made this way, overshadowing other routes to small peptide production. In fact, it is only since the discovery of the biosynthetic pathway for amanitin in 2007, that the ability of fungi to produce ribosomally synthesised and post-translationally modified peptides, known as RiPPs, was realised [4]. Since then a number of previously classified non-ribosomal peptides have been re-evaluated to demonstrate a rise in the number of fungal metabolites formed directly from conventional peptides by peptide maturation, also referred to as post-ribosomal peptide synthesis (PRPS; Fig. 1) [5].

**Fig. 2:**
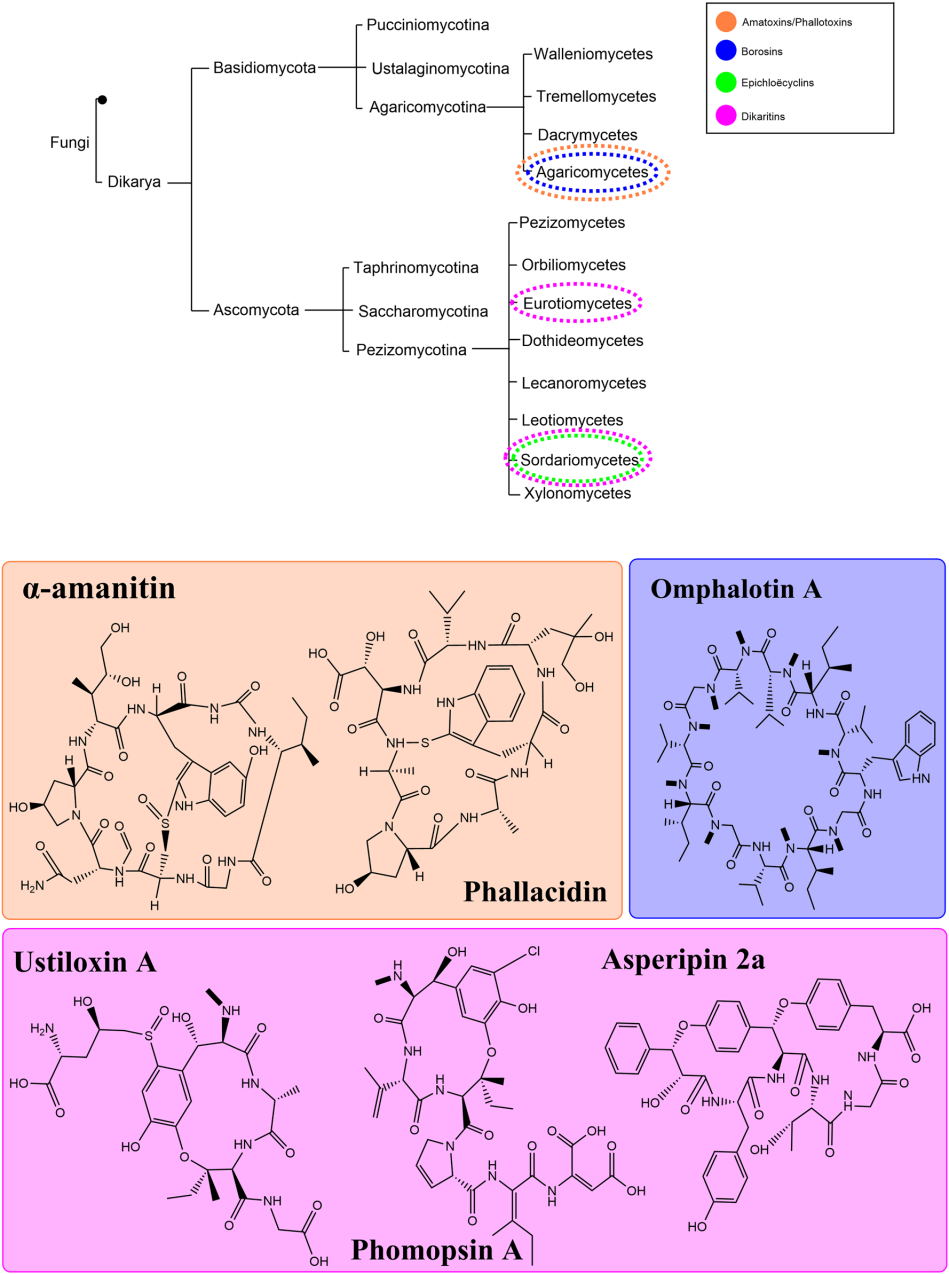
Simplified fungal phylogeny highlighting the classes which contain RiPP-producing species. Colours are used to identify which class of fungal RiPPs are produced in each instance. The chemical structures of key RiPP examples from these classes are shown using the same colour code

This delayed identification of fungal RiPPs (Fig. 2) is surprising given that such peptides from bacteria and animals have been exploited for many years. Bacterial RiPPs were often identified from screens for antimicrobial and antiviral activities [6–10]. Since the early 1950s we have been utilizing the lantibiotic nisin commercially for food preservation (Reviewed by Cotter et al. [11]). In terms of animal RiPPs, ziconotide from the marine snail *Conus magus*, was licensed to provide pain relief in the USA in 2004 [12]. Given that novel drug discovery is often the primary reason for exploring non-human peptides, investigations into newly classified and detected fungal RiPPs are somewhat limited in their scope. This is further exacerbated by the assumption, especially relating to fungal antibiotics, that our use of the compound is the same as the natural use by the fungus. So, fungal metabolites, including RiPPs, are subject to analysis under the anthropocentric lens. Even well-characterised metabolites such as penicillin often have very limited data on ecological purpose.

This approach, as explained by Li and Rebuffat [13] in their review of bacterial RiPP ecology, has stalled the development of knowledge regarding the purpose of RiPPs in nature, and how they may shape ecological communities—their chemical ecology [14, 15]. Spiteller [16] also noted that there was limited research conducted or interpreted from a fungal perspective. Whilst laboratory investigation has allowed functions to be assigned to fungal RiPPs, recent comparisons of the laboratory confirmed versus ecological roles of select antimicrobials have demonstrated that the purpose of a given metabolite in nature may differ substantially from the activity identified through laboratory tests [17]. This is especially true for those performed under ecologically unrealistic conditions and highly inflated concentrations [17]. This emphasizes the need to evaluate current knowledge of the ecological roles of fungal RiPPs. In light of the limited work conducted specifically into RiPP chemical ecology, in this review, the known activities of fungal RiPPs resolved from laboratory investigations will be considered in the context of the producing fungus’ natural environment. In turn facilitating predictions relating to the ecological purposes of these peptides.

## Current knowledge

### Basidiomycete RiPPs

#### Amatoxins and phallotoxins

Amatoxins and phallotoxins are bicyclic octa- and hepta-peptides, respectively [18]. The structures of amatoxins and phallotoxins have been known for many years, long before their synthetic pathways were understood as being peptide derived [4]. They are the major toxins of fungi such as *Amanita phalloides*, the infamous Deathcap fungus and so have long been a subject for research.

The biosynthesis of these starts with production of a conventional peptide (protoxin), but this is then extensively processed. Initially a prolyl oligopeptidase (POP) removes the 10-amino acid leader sequence of the protoxin, cutting after a conserved proline residue. This leader has the N-terminal amino acid sequence MSDIN, so the wider family have become known as MSDIN proteins. The same POP digests again after another proline, releasing the core of the toxin, which cyclises to give the core amino acid ring, typically of 7- or 8-amino acids [19, 20]. This is followed by cross-linking between the side-chains of a conserved tryptophan and cysteine, giving a bridge across the ring structure, and further modifications such as epimerisation of certain core amino acids. The genes encoding these enzymes are typically co-located as a gene cluster, however the high intron-density in these basidiomycete fungi can make their identification challenging without transcriptome data.

The amatoxin and phallotoxin RiPPs have been subject to intense research due to the human toxic effect of these peptides. The toxicity of amatoxins, is a product of their inhibition of the eukaryotic RNA polymerase II [21–23] as amatoxin binding to the enzyme changes its conformational state such that nucleotide incorporation and translocation is restricted [24, 25], preventing mRNA elongation and subsequent protein synthesis. As a result of this immense disruption, cells disturbed by the toxins begin apoptosis or necrosis [18]. Alternatively, but equally destructive, the phallotoxin mode of action is achieved through the toxin binding to F-actin, inhibiting its conversion to G-actin [26]. This F-actin stabilisation distorts the equilibrium of actin forms within the cell and as a consequence normal cell functions are disrupted [27]. The impact of phallotoxin on cell function can be seen specifically in its interference in cytoskeletal function [28] which, through cell membrane impairment, results in cell death [26, 29].

Regardless of mode of action, these RiPPs are undoubtedly capable of producing lethal effects and, as a consequence, steps have been taken to identify the fungi able to produce these toxins, with at least 35 species recognised [30], spanning four Basidiomycete families within the order Agaricales; the *Amanita*, *Galerina*, *Conocybe* and *Lepiota* [31, 32]. In contrast, phallotoxins are comparatively limited in their range of producing species, almost exclusively synthesised by species belonging to the *Amanita* genus [33], however exceptions to this rule exist [34]. Nonetheless, amatoxin- and phallotoxin- generating species are to a large extent united by their existence as wood-rotting fungi [31, 35–37].

#### Borosins

The borosins group is exemplified by the omphalotins [38]. Produced by the fungus *Omphalotus olearius* [38, 39]*,* omphalotins were discovered during a search for fungal metabolites showing nematocidal activity against *Meloidogyne incognita* [39]—a plant parasitic nematode capable of targeting at least 1098 species [40–43]. Examination of the omphalotins revealed their extremely selective nature towards *M. incognita* [44], albeit with reduced specificity at increased concentrations [45]. Originally omphalotins were believed to be produced by an NRPS, however such a gene could not be identified within the genome sequence of *O. olearius*. Instead the sequence WVIVVGVIGVIG, which is the core of omphalotin, was found to be encoded with the C-terminus of a gene annotated as a methyl transferase. Given that omphalotin has nine N-methylations, it was feasible that the toxin and the methyltransferase activity were derived from the same polypeptide. It was shown that the methyltransferase self-methylates within the toxin region [38, 46]. The methylated protoxin then undergoes cleavage, using a cluster-encoded prolyloligopeptidase (so similar to the amanitin and phalloidins above), removing the leader methyltransferase region, and then cyclising the core of the toxin.

Based on this gene arrangement of co-located N-methyltransferase and POP, the detection of comparable peptides and possible borosin RiPPs from other fungi has been permitted [38, 46]. Bioinformatic analyses have facilitated greater comprehension of the vastness of the borosin category with Quijano et al. [47] uncovering 50 novel putative RiPP gene clusters. Of the proposed RiPPs, the gymnopeptides [47], from *Gymnopus fusipes*, with anti-proliferative effects on human cancer cells [48], as well as the lentinulins and dendrothelins from *Lentinula edodes* (shiitake mushroom) and *Dendrothele bispora*, respectively [49], have been confirmed as borosin peptides. Given that the borosins gene clusters also commonly include other genes typical of those involved in further tailoring of natural products, this family of structures is likely to be larger still. Recent work by Miller et al*. *[50] revealed the presence of “split borosins” in bacteria, and so this is the only RiPP family so extensive that it spans both fungal and bacterial domains of life.

### Ascomycete RiPPs

#### Dikaritins

Three RiPP representatives groups have been confirmed within the dikaritins; the phomopsins [51], ustiloxins [52] and asperipins [53]. Akin to the amatoxins/phallotoxins and borosins, the functions of the dikaritin RiPPs are generally understood thanks to their investigation prior to their classification as RiPPs. The discovery of phomopsins resulted from an investigation into the causative agent of lupinosis, a liver disease that develops following the consumption of lupins (*Lupinus spp.*) [54]. Experimental analyses, however, revealed lupinosis to be instigated by the ingestion of compounds from the lupin pathogen *Phomopsis leptostromiformis* [55] rather than the lupin plant [56, 57]. Searches for the specific *P. leptostromiformis* metabolite responsible for lupinosis were undertaken, leading to the isolation and characterisation of phomopsins A and B [58], antimitotic mycotoxins which exert their affect through tubulin binding and the inhibition of the spindle formation essential to mitosis [55]. It is this factor that even prior to their classification as RiPPs led to comparisons being drawn between phomopsins and the ustiloxins [59, 60]. The ustiloxin peptides (A and B) are known to be produced by *Ustilaginoidea virens* [59] and *Aspergillus flavus* [52, 61, 62]—though ustiloxin B alone is synthesised by *A. flavus* rather than the full complement of ustiloxin peptides—and these too have an antimitotic function [60]. Indeed, the ustiloxins also interact with tubulin to inhibit microtubule assembly [59, 63] and given this similarity, the ecological functions of the metabolites are likely to be similar.

The dikaritins are synthesised from a repetitive protein that typically contains multiple copies or variants of the core toxin. The protoxin is targeted to the golgi where it undergoes Kex2 mediated proteolysis, coupled with further processing by cluster-encoded peptidases to release each toxin-core. These then undergo multiple modification. For the ustiloxins and phomopsins this involves crosslinking the side-chain of the N-terminal tyrosine to that of an internal isoleucine within the core, cyclising the molecule, then further processing such as oxidations or methylations. This cyclisation is performed by enzymes with homology to UstYa and UstYb of the DUF3328 family.

The initial processing of the dikaritins bears striking similarity to the processing of the *Saccharomyces cerevisiae* α factor mating pheromone where a multicore precursor peptide is targeted to the Golgi apparatus and initial processing is undertaken by Kex2 proteases [64]. This raises the question of whether the origin of such RiPP peptides stems from duplication and modification of mating pheromone peptide genetic clusters.

The asperipins are currently grouped within the dikaritins. They were identified from bioinformatic searches for other gene clusters that contained a repetitive protoxin gene, co-located with homologues of UstYa and UstYb, the enzymes responsible for the cross-linking of the tyrosine side chain in ustiloxin biosynthesis. In asperipin however, the toxin is not cyclised by linkage between an N-terminal tyrosine and an internal isoleucine, but with two linkages from internal tyrosine side-chains, forming a novel bicyclic core [53], so may well be classified as a new family of fungal RiPPs.

It has recently been shown that victorin—the host-specific toxin of *Cochliobolus victoriae* impacting the Victoria cultivar of oats, is also a RiPP rather than NRPS product [65]. As with the other dikaritins, the multi-core protoxin is likely processed by Kex2 as well as gene-cluster-encoded peptidases and undergoes extensive modification after cyclisation, but in this case it is cyclised between the C-terminal phenylalanine side chain and an internal leucine. Unusually for fungal products, victorin is chlorinated as part of its maturation.

#### Epichloëcyclins

Of the known fungal RiPP classes, the most recent is the epichloëcyclins found from grass-endophytic fungus *Epichloë sp.* These were identified from a fungal transcript that was abundantly expressed in planta, encoding a repetitive protein (GigA) with a golgi-targetting leader sequence. Comparison of LC–MS traces from from apoplastic fluids of grasses colonised by the wild-type and *GigA* deletion mutant, allowed identification of oligopeptides that were absent in the mutant-derived extracts. These corresponded with the core repeats of the GigA peptide, giving epichloëcyclins A-F, cyclic nonapeptides generated as a result of imperfect amino acid repeats within the multicore RiPP precursor protein, encoded by the *GigA* gene [66]. Like the dikaritins, the protoxin is likely to be Kex2 processed, and the cores cyclised by cross-linkages between tyrosine and isoleucine side-chains, however in this case the isoleucine is N-terminal and the tyrosine internal to the core. The functions of each of these peptides are unknown [67] but by investigating the ecology of epichloëcyclin-producing fungal species, the *Epichloë* endophytes, the ecological roles of these RiPPs may be predicted, using this to guide future research. *Epichloë* endophytes are capable of forming a range of associations with host plants, namely grasses belonging to the *Pooideae* subfamily [67, 68], varying from parasitic to mutualistic [67]. While in these relationships, epichloëcyclin metabolites are synthesised abundantly by the fungus, significantly altering the plant apoplast metabolome as the secreted fungal peptides enter this space [66, 67]. Reasonably, this has led researchers of these RiPPs to investigate the role of the peptides in host-fungal communication.

## Chemical ecology

In common with conventional small metabolites there is a plethora of different roles the RiPPs may perform. Given that small peptides could be made by a RiPP or an NRPS and result in very similar structures there is likely to be considerable overlap in ecological functions.

### Defence

Production of defensive compounds is a tactic commonly employed by organisms which are incapable of locomotion and therefore require an alternative means of protection from consumers and competitors to fleeing. The inability to rapidly escape the threat of consumption consigns fungi to producing compounds that are distasteful or toxic, such as protease inhibitors [69]. Here, given fungal ecology and laboratory identified toxicity, the action of select RiPPs in this role is examined.

#### Intentional mycophagy

**Fig. 3:**
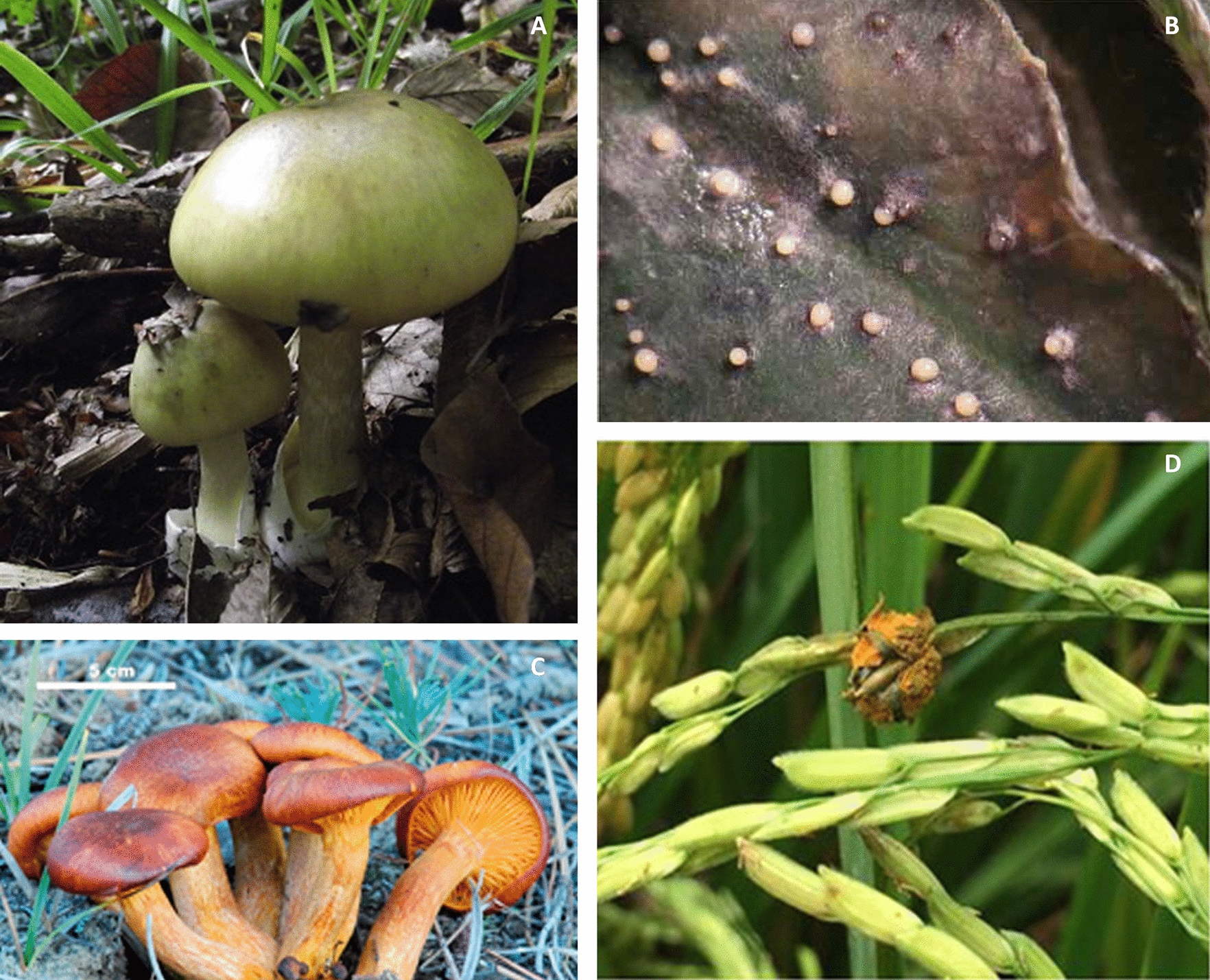
Fungal RiPP-producers with presumed roles in defence. **A** *Amanita phalloides* [70], **B** A lupin leaf showing symptoms of *Phomopsis leptostromiformis* infection [71], **C** *Omphalotus olearius* [72], **D** False smut disease symptoms on rice following *Ustilaginoidea virens* infection [73]. Permission to reproduce figures from the publishers of Kaya et al*.* (2013) (Elsevier); Bal et al*.* (2016) (Taylor & Francis), Lin et al*.* (2018) (Springer Nature) and Cowley et al*.* (2012) (Taylor & Francis)

As noted previously, most amatoxin- and phallotoxin-generating species are wood-rotting fungi [31, 35–37] and since Hutchison et al*.* [74] argue that fungi in decaying woodland environments represent a high nutrient food resource for foragers, an evolved function of amatoxin/phallotoxin RiPPs in defence should be considered. This role is implicated by the localisation of amatoxins and phallotoxins to the cytoplasm [75], rather than their secretion, as the use of these toxins against organisms which present a threat to fungal survival beyond the risk of consumption, such as a competitive threat, would rely on secretion of the toxic peptide into the environment in order for it to take effect. In addition, since amatoxin and phallotoxin producing species are often mushroom forming, to be truly involved in the prevention of consumption, it is expected that the location of greatest toxin concentration within the fungus would be in the pileus as this is most visible to foragers (Fig. 3A). Accordingly, amatoxin and phallotoxin concentration is greatest in the fruiting body and gills of the mushroom [76, 77] which would be detected and targeted far more frequently than small hidden hyphae [76]. Thus, this localisation is evolutionarily advantageous to the fungus.

The ecological use of amatoxins in deterring mycophagy, has been confirmed by several experiments and case studies, with the amatoxin peptides inducing severe toxic effects in insects, nematodes, and mammals alike [75], exerting their influence primarily on the digestive systems of these organisms following ingestion of the fungus. In mammals this translates to organs such as the liver and kidney being worst affected [21, 78]—hepatic and renal failure are often cited as the leading cause of death in human cases of amatoxin poisoning [79]. Hence, amatoxin RiPPs do appear to have evolved in response to the threat of consumption by eukaryotic organisms.

The presence of similar characteristics in other RiPP producing species may too be indicative of a defensive function. Consumption of the fruiting body of the gymnopeptide-producing species, *Gymnopus fusipes*, can also induce gastrointestinal symptoms, despite general acceptance that these fungi are edible [48] and so the defence hypothesis may also be applied here. It is worth mentioning though that due to the medical focus on these compounds basic data on tissue localisation is missing for many RiPPs with the research focus being on lab-based fermentation. Indeed, the gymnopeptides have largely been studied in the context of cancer, though the anti-proliferative influence of the metabolites lies in their cytotoxic activity [48] which would be effective against all eukaryotic cells. This is a typical example of how the investigated function of a peptide may differ from its ecological function with Kunzler [80] arguing that cytotoxic secondary metabolites have mainly evolved for fungal defence against arthropods. However, this role for the gymnopeptides is yet to be verified.

Importantly, it must be noted that though toxic, the ecological purpose of phallotoxins remains elusive, since phallotoxins only induce toxicity when administered parenterally [18, 81]. Low absorption of the toxin when ingested orally [4] has led researchers to conclude that the human toxic effects of phallotoxins are limited [77]. However, this is an undeniably anthropocentric view since in nature the fungus cannot inject and administer the toxin into fungivores. It is then difficult to understand how a toxic effect through injection could evolve. Instead, we must appreciate that a lack of human toxicity through ingestion does not mean phallotoxins are degraded by the digestive systems of all organisms. For smaller organisms, such as insects and nematodes, a high dose of phallotoxin ingested orally could prove fatal [82] but research focussed on mammals may have ignored this.

#### Unintentional mycophagy

In addition to intentional mycophagy, where the fungus itself is the desired nutrient resource, unintentional mycophagy also exists owing to the nature of many fungi as plant pathogens and parasites. By residing in, on, or in the proximity of, desirable plant resources, consumption of these fungi can occur. This is perhaps best considered in the context of the omphalotins where the target of RiPP toxicity is the plant parasite *M. incognita,* rather than a mycophagous organism. Yet, the widespread prevalence of the parasite, which penetrates host plants through the root system [39], and the existence of *O. olearius* on tree roots [72], means that contact between the two species is highly likely. As such, it is plausible that the nematocidal activity of omphalotin evolved to mitigate the ecological impact of the nematode on the fungus. Since omphalotin is not secreted from the fungus into the surrounding environment [39], the toxic action of the peptide may only be realised if the fungus is consumed, once again indicating a RiPP’s function to be in defence against mycophagy. In contrast to the basidiocarp localisation of RiPPs in the majority of amatoxin-producing species [45, 76, 77], the omphalotin peptide is located in the fungal mycelia rather than the fruiting body of the fungus [47, 83] despite its high visibility (Fig. 3C). This variance in RiPP localisation is likely attributable to differences in the threat presented, as in this fungus-nematode interaction, it is the *O. olearius* hyphae which are at risk of consumption as they exist in an environment being invaded by nematode parasites. Here, the likelihood of unintentional mycophagy is high. Lentinulin, from *Lentinula edodes*, also has nematocidal activity and is localised to the mycelia [49]. As such this peptide may equally play a role in deterring unintentional mycophagy from alternate plant pests.

Correspondingly, other plant pathogenic RiPP-producers also display toxicity when consumed, with lupinosis symptoms, which develop following the consumption of *P. leptostromiformis*—having greatest impact in the liver of mice, rats and sheep [55]. Equally, cases of ustiloxin consumption via ingestion of the *U. virens* rice plant host, have also resulted in animal poisonings [59]. Again, these fungi are unlikely to be the target of consumers since they are often far less visible than their hosts (Fig. 3B, D). Indeed, in *U.virens* infection symptoms only appear following host flowering [84], while in *P. leptostromiformis* infection, symptoms are usually minimal, causing only small lesions on lupin stems and pods [85]. As a result, it is more feasible that the host plant is the desired resource for grazers, rather than the fungus residing within it. Accordingly, it is probable that both the ustiloxin and phomopsin mycotoxins have evolved due to high levels of unintentional mycophagy. For phomopsin this theory is further supported by the knowledge that phomopsin synthesis appears to be instigated by moisture [55, 57], when tough-stemmed lupin plants become more palatable to herbivores [57], allowing the coincidence of phomopsin production with a period of increased threat.

### Competition

In many instances, the ecological functions of RiPP chemicals are two-fold as the compounds evolve from the threat of unintentional mycophagy but also afford plant pathogenic RiPP-producers a competitive advantage over other organisms with an affinity for the same nutrient source. Since these fungi are often consumed alongside their host plants, the toxic RiPPs facilitate fungal monopolisation of the plant by converting it to inedible material while removing competitors through poisoning. Even if sublethal, it is likely that grazers develop an association between the consumption of the host plants of the fungal pathogens responsible for RiPP production, and mycotoxin poisoning. Hence, through associative learning, it would be expected that grazing of these host plants becomes reduced, as consumption of the host becomes synonymous with poisoning from RiPPs. This leaves greater opportunities for nutrient acquisition open to the fungi. Consequently, production of toxic RiPP compounds would directly influence the prevalence of the plant pathogens, therefore, the importance of the peptides to producing-fungi becomes appreciable.

### Movement

As the ecological roles of RiPP compounds are largely speculative, some RiPPs have several plausible and often unrelated proffered roles with the most drastic contrast in possible functions found in the phallotoxins. Analyses of these compounds have shown them to be mycotoxic, yet as this toxicity only occurs following injection, it may instead be the case that this identified activity is nothing more than a by-product of the ecologically unrealistic concentrations of the peptide investigated experimentally. To understand its chemical ecology, the putative roles of the RiPP at reduced quantities must be considered. Notably, as phallotoxin influences cytoskeletal function, the RiPP could alternatively be used by the fungus to control its own cell growth and by extension locomotion [86]—since fungi move by growing in a given direction rather than locomotion in the truest sense. As some phallotoxin producing species have been shown to vary in their phallotoxin concentration depending on ecology [87] it follows that fungi may manipulate their own phallotoxin production through selective gene expression [76] to reach favourable environments. Nevertheless, this hypothesis is yet to be substantiated as studies investigating the influence of phallotoxins on cell growth have largely used mammalian rather than fungal cells. Though these are both eukaryotic cell types, and therefore should be similarly affected by toxin application, it is unknown whether the producing fungus has resistance to its own peptide.

### Nutrient acquisition

The role of RiPP peptides in fungal nutrient acquisition has long been an area of interest in RiPP research with this hypothesis first proposed for ustiloxin, since the peptide appears to have a toxic function against plant in addition to animal cells [59, 88], causing swellings in roots of rice seedlings [60, 89]. However, as different *U. virens* isolates vary in their production of ustiloxin, with some isolates which lack the peptide entirely still displaying symptoms of phytotoxicity but unable to cause animal toxicity [90] the primary purpose of the ustiloxins appears to be in animal deterrence with any effects on plant cells being secondary.

A better example is perhaps that of victorin. This is a well-characterised host-selective toxin of oats carrying the *Vb* gene. Originally believed to be made by NRPS, victorin has recently been demonstrated to be a RiPP [65], which is essential for plant pathogenicity through stimulating premature leaf senescence, a function without which the producing-pathogen is avirulent [91].

The victorin RiPP-producer, *Cochliobolus victoriae*, is a necrotrophic phytopathogen of oats [92]. The RiPP likely facilitates increased nutrient acquisition as it transforms living material into a form of use to the fungus. The toxin achieves this by binding two mitochondrial glycine decarboxylase complex proteins, cleaving the large Rubisco subunit, and as a result inhibiting the plant’s photorespiratory cycle [91]. As such, this toxin is instrumental in the establishment and survival of the fungus—provided the host oat plant is of the ‘Victoria-type’ [93] and contains the *Vb* gene which determines sensitivity to the toxin and by extension the pathogen [94]. Indeed the importance of victorin extends beyond its role in plant pathogenicity due to its significance in developing the concept of host-selective plant toxins [91]. Exploration of this RiPP therefore highlights how understanding the ecological role of these peptides can hold value beyond academic interest.

### Symbiotic associations

In contrast to the function of the victorin RiPP in triggering plant senescence, epichloëcyclin RiPPs have been implicated in plant-fungi symbiosis based on the abundant expression of *GigA* from fungi in endophytic associations, and the absence of these transcripts in fungal culture (A. Koulman, G. Lane, unpublished data; 67, 75)—potentially facilitating communication between the two organisms. Yet, as deletion of the *GigA* gene has no great phenotypic influence on the plant with which the fungus is associated [67], if the role of the peptide is in communication, the effect does not appear to be significant. It has however been noted that in some mutualistic plant-fungi interactions, fungi serve to benefit the host plant through performing a protective function against other organisms [95, 96] as well as the biotic environment [97, 98]. Therefore, it may be of interest to conduct investigations into the influence of these fungal peptides during host stress, as despite no obvious functions having been uncovered through gene knockouts, these tests were, to our knowledge, conducted under stress-free conditions [67]. Thus, it is plausible that the production of these RiPPs in combination with plant stress signals is responsible for triggering the fungus’ protective action. Until these stress-related tests are completed it would not be possible to detect a fungal role in plant protection during symbiosis. If found to aid the survival of select plant species, the fungi, through the action of these peptides, would undeniably shape their immediate surroundings. This manipulation of ecology would be for the benefit of the fungus as they ensure the survival and propagation of a host that enables profitable associations. As the *Epichloë* fungus itself may be vertically transmitted alongside infected plant seeds [99] it becomes apparent how fungal facilitation of plant survival could serve to the endophyte’s advantage as it becomes synonymous with the survival of the *Epichloë* fungus itself.

## Chemical ecology-based applications

Several of the RiPPs discussed within this review have already been subject to commercial, scientific, and medical applications, however, many of these are unrelated to chemical ecology. Yet, by ignoring these functions, alternative uses of these peptides remain unexplored. As such, plausible chemical ecology-based applications are proposed here.

### Antifeedants/pesticides

Based on the toxicity of several fungal RiPPs against plants and animals, these peptides may be useful as a novel source of agrochemicals, effective against organisms that threaten plant health and crop productivity. This is not a possible application for all toxic peptides, we must remember that though amatoxins/phallotoxins repel mammalian pests from consuming crops so too would they have this effect on humans. Therefore, there is a requirement to investigate the selectivity of mycotoxins prior to applying them in an agricultural setting. Nevertheless, since RiPPs with toxicity against plants [65], arthropods [80], nematodes [39] and mammals [59, 100] are all known or presumed to exist (pending functional confirmation) it follows that multiple treatments based on these RiPPs may be developed with each targeting the specific pests against which the peptides are active. Indeed, the agrochemical application of omphalotins has already been proposed [101]. Importantly, the borosin and dikaritin RiPPs, as well as the victorin peptide, are all produced by fungi which parasitize plants, therefore any initiative to utilise these peptides directly would require the extraction and purification of these metabolites, allowing potential pesticidal benefits to be realised without the losses associated with fungal infection. Hence, these RiPPs might be exploited to increase plant productivity directly, or used to inspire development of synthetic agrochemicals.

### Protection from abiotic stress

In addition to pests and pathogens, plant productivity is influenced by abiotic factors and limited by stresses such as extreme weather temperatures, drought and nutrient deficiency. *Epichloë* fungi, when associated with a host plant, can increase pasture persistence and productivity [68]—with this knowledge already being utilised commercially. However, it is still unknown whether the epichloëcyclin RiPPs play a role in this. Should future investigations, performed under stress conditions, confirm a protective function of the RiPP, the applications of this interaction are already being realised. However, knowledge of the mechanism underpinning such associations may aid in the development of next generation commercial endophytes [68] and facilitate the extension of this beneficial association to alternative plants including crop species.

### Novel RiPP discovery

Perhaps most significantly, knowledge of RiPP chemical ecology may accelerate novel RiPP discovery. A holistic approach to looking at RiPPs in the context of their fungal-producer, and its environment, may prove to be exceedingly insightful. Since fungal ecology is instrumental in determining the fate of peptides, pushing them in specific evolutionary directions, comprehensive investigations may enable the identification of the environmental conditions conducive to generating RiPPs with specific properties and functions which may be of use to mankind. For instance, based on the ecology of amatoxin producing fungi as wood-rotting species, it is probable that RiPPs from other fungi in similar environments will fulfil comparable ecological roles as similar evolutionary pressures for development of an antifeedant are experienced. New RiPPs with novel functions are continually being discovered with victorin being the most recent example of this. Importantly, knowledge that RiPPs may exist as virulence factors promotes research into the development of suitable plant disease control methods based on known RiPP biosynthetic mechanisms and as such the wider implications of RiPP research on food security are beginning to emerge.

## Conclusion

This review summarises the known RiPP ecological functions to date, with the majority aiding in defence from various potential predators, and offers suggestions for applications of RiPP peptides based on these roles. Not only does this highlight the value of considering fungal chemical ecology in novel metabolite analysis, but also substantiates why RiPP research is an area of current relevance and importance. The continued advancement of fungal RiPP knowledge related to function is especially pertinent as computational techniques, specifically genome mining, facilitate the discovery of an ever-increasing number of novel RiPP clusters and peptides [102–106]. This has been true for bacterial RiPPs, allowing identification of further lanthi- and thioamidated peptides [107–109] but as seen in work by Quijano et al*.* [47] fungal RiPP research is now following suit. Though insightful, peptide discovery alone does not allow the realisation of the benefits of RiPPs or exposes the extent of peptide toxicity. Future RiPP research will therefore be maximised in terms of discovery, analysis, and applications if a three-pronged approach utilising bioinformatic, chemical, and ecological analyses is applied.

Both RiPPs and NRPS-derived small peptides share common properties, hence the confusion about their origins. They are typically small cyclic peptides that are recalcitrant to digestion. They are mutable in so much as they can exist as families of related sequences, whether this is achieved by having multimodule RiPPs with differing core sequences, expanded gene families as seen in the MSDIN amatoxins, or via NRPS with reduced module specificity so delivering a cocktail of similar compounds. They can both include residues not normally associated with proteinogenic aminoacids, be this by direct incorporation in NRPS, or by post-translational modification in RiPPs. These similar outcomes are achieved through completely separate routes, and such convergent evolution suggests that these types of molecule are important in ways that we are only just beginning to appreciate. Their ecological roles are likely to emerge as we learn more about these classes of fungal RiPP. We would expect many more RiPPs to be discovered in the future and further classes may emerge as fungal proteomics is better understood.

## Data Availability

Not applicable.

## Abbreviations

RiPP: Ribosomally synthesised and post-translationally modified peptide
PRPS: Post-ribosomal peptide synthesis
